# Intracoronary Imaging for the Management of Vulnerable Plaques

**DOI:** 10.3390/jcm15051678

**Published:** 2026-02-24

**Authors:** Francesco Maria Animati, Rocco Antonio Montone, Francesco Fracassi, Luigi Cappannoli, Andrea Caffè, Vincenzo Scarica, Francesco Burzotta

**Affiliations:** 1 Dipartimento di Scienze Cardiovascolari e Pneumologiche, Università Cattolica del Sacro Cuore, 00168 Rome, Italy; francescomaria.animati01@icatt.it (F.M.A.); andreacaffe97@gmail.com (A.C.); vincenzo.scarica01@icatt.it (V.S.); francesco.burzotta@unicatt.it (F.B.); 2 Department of Cardiovascular Medicine—CUORE, Fondazione Policlinico Universitario A. Gemelli IRCCS, 00168 Rome, Italy; francesco.fracassi@unicatt.it (F.F.); luigi.cappannoli@gmail.com (L.C.)

**Keywords:** optical coherence tomography, intra-vascular ultrasound, vulnerable plaques, thin-cap fibroatheroma, percutaneous coronary intervention

## Abstract

Vulnerable coronary plaques are the primary substrate for acute coronary syndromes, representing a significant challenge in cardiovascular care. This review examines the pivotal role of intracoronary imaging, specifically intravascular ultrasound (IVUS) and optical coherence tomography (OCT), in the detection and management of these high-risk lesions. We detail the technical principles of these modalities and their unique capabilities in characterizing plaque morphology, from identifying thin-cap fibroatheromas (TCFA) to differentiating mechanisms of plaque rupture and erosion. The article further synthesizes evidence on how imaging-guided strategies—ranging from intensive medical therapy to emerging interventional approaches like preventive stenting—can stabilize vulnerable plaques and improve patient outcomes. Finally, we explore future directions, including the integration of artificial intelligence and hybrid imaging technologies, which promise to refine risk stratification and personalize the treatment of coronary artery disease.

## 1. Introduction

Coronary angiography is the diagnostic gold standard for coronary artery disease, localizing plaque position and quantifying the associated stenosis, and the primary guide for percutaneous coronary intervention (PCI). However, angiography provides only a two-dimensional luminogram and cannot completely characterize plaque composition or confirm optimal stent apposition [1,2].

**Figure 1:**
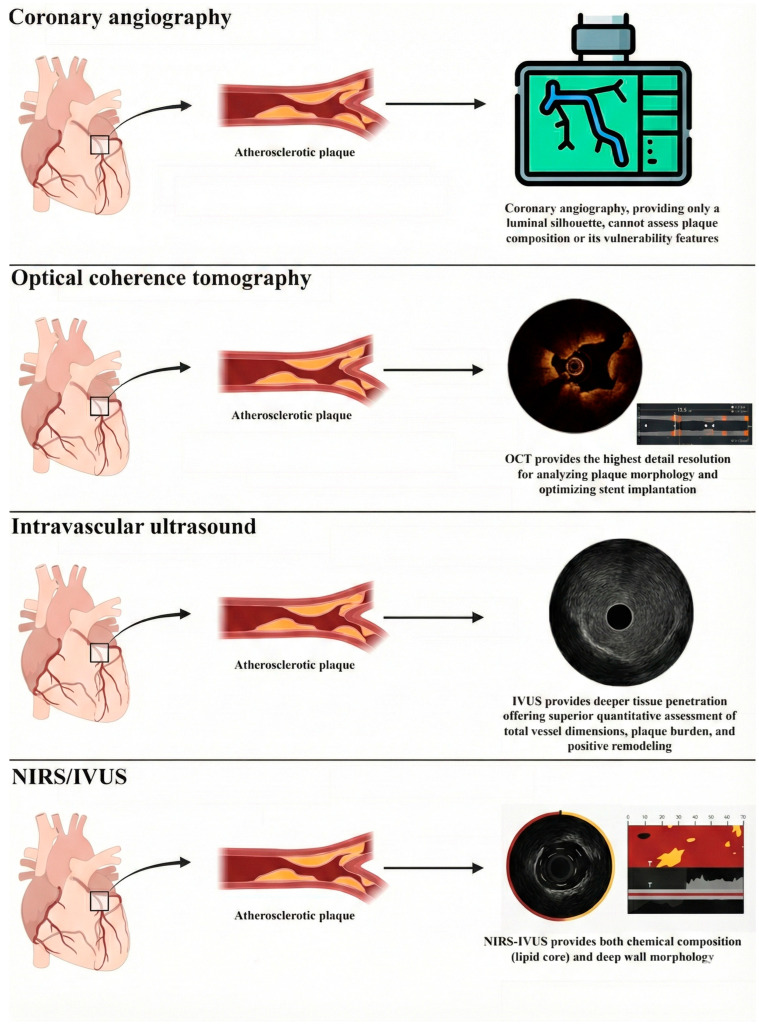
Provides a summary of the principal features of intracoronary imaging techniques, specifically about ICI tissue characterization capabilities. Abbreviations: OCT = Optical Coherence Tomography; IVUS = Intravascular Ultrasound; NIRS/IVUS = Near-Infrared Spectroscopy/Intravascular Ultrasound.

The main contemporary intracoronary imaging (ICI) modalities are intravascular ultrasound (IVUS), optical coherence tomography (OCT), and near-infrared spectroscopy (NIRS), and they all supplement angiography by delivering high-resolution, cross-sectional images from within the vessel lumen (Figure 1; Table 1) [1]. Of note, the three available ICI modalities have different operating principles that consequently generate device-specific coronary plaque characterizations. All of them share a clear superiority over angiographic images alone, and this created great expectations regarding their potential value to help understand the physiopathology underlying acute coronary syndromes (ACS) and identify plaque vulnerability [3,4]. With regard to the latter, the concept of “plaque vulnerability” has its roots in the late 1980s, when it was first hypothesized that a vulnerable plaque was a plaque characterized by an elevated propensity for rupture and for causing ACS [5]. At present, however, the designation “high-risk plaque” is considered more appropriate, as it encompasses the precursors of the three principal substrates known to trigger luminal thrombosis, namely plaque rupture, plaque erosion, and eruptive calcified nodules [6,7,8]. Thin-cap fibroatheroma, characterized by a large lipid-rich core and an overlying thin fibrous cap, has represented for decades the prototypical vulnerable plaque. Nowadays, some other features of vulnerability have been found to be also relevant: a lipid-rich necrotic core, a large plaque burden, active plaque inflammation, endothelial damage, intraplaque hemorrhage, positive remodeling, neovascularization, and superficial protruding calcified nodules represent only some of the features that define a plaque as a high-risk one [6]. The identification of these plaque features is therefore crucial for guiding treatment strategies and for determining whether a patient is more suitable for conservative management or for an invasive approach and which type of invasive strategy should be pursued [9,10] As these features can be adequately assessed only by intracoronary imaging (ICI) techniques, this underscores the pivotal role of ICI in contemporary interventional cardiology.

## 2. Intracoronary Imaging Techniques

**Intra-vascular ultrasound (IVUS)**

The idea of imaging vessel walls from inside via ultrasound emerged in the early 1970s. In 1972, Bom and colleagues first introduced an IVUS transducer capable of generating cross-sectional images without mechanical movements [13]. These transducers were primarily used to diagnose atherosclerosis and to monitor stent placement procedures. First prototype systems date back to the 1980s, when Dr. Patrick Yock’s group built the first catheter-based IVUS consoles and flexible probes. These laboratory prototypes adapted high-frequency ultrasound (30–40 MHz) to fit within coronary guidewires, allowing for cross-sectional imaging of lumen and wall structures for the first time [14]. In 1988, Yock et al. managed to accomplish IVUS first in vivo human use, demonstrating that IVUS catheters could safely navigate coronary arteries and produce real-time, 360° grayscale images of plaque burden and vessel morphology, capabilities that angiography alone could not reveal [1,14]. Over time, IVUS evolved from a single-element rotating design to high-resolution, electronically steered phased arrays. Modern systems integrate 3D reconstruction, co-registration with angiography, and automated plaque quantification, transforming IVUS from a research tool into the clinical adjunct for planning, guiding, and assessing percutaneous coronary interventions, which is nowadays widely known [15].

**Optical coherence tomography (OCT)**

Optical coherence tomography (OCT) was first presented in 1991 by Professor James C. Fujimoto from the Massachusetts Institute of Technology (MIT), who was also the first to publish OCT imaging results on biological tissue [16]. Since then, OCT emerged in many fields of medicine as a powerful tool able both for basic research and clinical applications. Although the first in vivo use of OCT was achieved in the field of ophthalmology and dates back to 1993 [17], the potential of OCT in cardiology was evident from its earliest applications [18].

In 2000, Tearney et al. demonstrated the feasibility of OCT in porcine models, successfully visualizing coronary structures and stents [19]. That same year, the Massachusetts General Hospital group, led by Professor Jang, performed the first human intracoronary OCT study, confirming safety and superior resolution compared with IVUS [20]. In 2002, Jang and colleagues validated OCT plaque characterization against histology in a large cadaveric study [21]. By 2005, OCT was applied in vivo to acute coronary syndrome, revealing a higher prevalence of thin-cap fibroatheromas in unstable versus stable patients [22]. Subsequent advances, particularly frequency-domain OCT, improved image quality, and usability, ultimately led to FDA approval in 2010 [23].

Since its higher resolution compared to IVUS, OCT became the first choice tool to evaluate plaque cap thickness and to characterize plaque phenotype in the culprit lesions of ACS (id est, to differentiate between plaque erosion, calcified noduli, and thin-cap fibroatheroma) [18,24,25].

From their introduction, IVUS and OCT have gained progressively more attention from interventional cardiologists and now are considered an indispensable tool for some technically difficult procedures (both diagnostic and therapeutic) and to identify high-risk plaques [6,26,27].

In accordance with this, they have been included in European Society of Cardiology (ESC) guidelines, both for chronic coronary syndromes and acute coronary syndromes [28,29]. As for chronic coronary syndromes, ESC 2024 guidelines recommend the use of IVUS to evaluate the severity of intermediate stenoses of left main stem prior to revascularization (Class of Recommendation IIa—Level of evidence B) [28].

As for ACS, ESC 2023 guidelines recommend the use of intracoronary imaging in cases of culprit lesion ambiguity or to guide PCI in clear culprit lesions [29].

In the first scenario, culprit lesions’ ambiguity—which occurs in over 30% of suspected NSTE-ACS cases—the use of intracoronary imaging—preferably OCT—and a subsequent imaging-based treatment is recommended (Class of Recommendation IIb) [29].

In the second case, for a clear and PCI-suitable culprit lesion coronary identified at coronary angiography, ESC ACS 2023 guidelines recommend the use of intracoronary imaging to guide intervention (Class of Recommendation IIa) [29].

**Near-infrared spectroscopy (NIRS)**

Near-infrared spectroscopy is a recently introduced intracoronary imaging technique now often used to assess vascular plaque. Initially applied in 1993 to measure lipid plaques in preclinical models, it was later validated in human cadaver studies and clinical series [30]. Although NIRS finds several applications in medicine, it has gained a prevalent role in coronary artery and vulnerable plaque evaluation [31,32]. More precisely, NIRS has been validated and approved by the FDA against the gold standard (i.e., histology) to detect and map lipid-rich plaques [33,34]. Since the main limitation of the NIRS technique lays in the impossibility to produce a three-dimensional image of the coronary artery as it delivers solely semiquantitative measurements [35], the latest NIRS platform integrates NIRS and IVUS into a single catheter, delivering simultaneous data on plaque composition and vessel structure within the coronary arteries [36].

**Other intracoronary imaging techniques**

- **NIRS/IVUS**

NIRS/IVUS employs near-infrared light and ultrasounds as its energy source, achieving a spatial resolution of up to 100 µm with a penetration depth ranging from 4 to 8 mm (Figure 1) [6,36]. NIRS/IVUS is emerging as a technique that offers supplementary insights about plaque compositions and mitigates the specific drawbacks associated with IVUS and OCT [37].

Histopathological validation shows that combining IVUS and NIRS significantly boosts both sensitivity and positive predictive value for detecting fibroatheromas compared to using either modality alone [38]. Specifically, the presence of either IVUS attenuation or NIRS lipid-rich plaque detection raised sensitivity to 63%, while combining both resulted in an 84% positive predictive value, substantially higher than either modality alone (~65–66%) [38].

The Lipid-Rich Plaque Study, a large prospective multicenter trial led by Waksman et al. [39], enrolled 1563 patients with coronary artery disease who underwent NIRS-IVUS imaging of at least two nonculprit arteries. At two years, patient-level analysis showed that each 100-unit increase in maxLCBI_4mm_ was associated with an 18% higher risk of nonculprit lesion MACE, while segments with maxLCBI_4mm_ ≥ 400 had an adjusted hazard ratio of 3.39 (95% CI 1.85–6.20; *p* < 0.0001). These findings demonstrate that NIRS-IVUS enables reliable risk stratification by identifying lipid-rich plaques predictive of adverse cardiac events, thereby highlighting its prognostic utility in clinical practice.

- **Emerging hybrid intracoronary imaging techniques**

Intracoronary imaging is rapidly evolving, with dual- and hybrid-probe catheters. By now, five dual-probe catheters have been used in cardiology studies: NIRS-IVUS, IVUS-OCT, OCT-NIRS, OCT-NIRF (near-infrared fluorescence), FLIm-OCT (fluorescence lifetime imaging OCT), and emerging FLIm-IVUS and IVPA IVUS (intravascular photoacoustic-IVUS), enabling combined structural, compositional, and functional assessments [40,41]. These innovations are moving from preclinical studies toward human trials, promising more comprehensive coronary evaluation.

Of particular interest is VH-IVUS (Virtual Histology Intravascular Ultrasound), an advanced intracoronary imaging technique that processes the radiofrequency backscatter signals from a conventional IVUS catheter using spectral analysis. By applying a classification algorithm to the decomposed signal, it reconstructs a color-coded tissue map, providing a virtual histology of the atherosclerotic plaque by identifying four primary tissue components: fibrous, fibro-fatty, dense calcium, and necrotic core [42,43].

Artificial intelligence (AI), particularly deep learning (DL), further enhances ICI by automating image analysis and reducing reliance on operator expertise [44,45]. In this context, OCT-based AI algorithms may facilitate automated quantification of key parameters, such as plaque burden and minimal lumen area (MLA), both essential for guiding pre-PCI decision-making and optimizing post-PCI outcomes [46]. By providing these measurements, AI can support operators in determining critical procedural aspects, including the appropriate landing zone, the minimum stent length required to cover the MLA, the minimal stent area (MSA), and the degree of stent expansion [46]. Moreover, OCT AI-based automated detection proved to be prognostically relevant also in identifying TCFA in patients with myocardial infarction. PECTUS-AI study [47]—a secondary analysis of the prospective observational PECTUS-obs trial [48] involving 438 myocardial infarction patients who underwent OCT of all fractional flow reserve–negative nonculprit lesions—showed that AI-based TCFA identification with OCT is a feasible option, requiring exclusion of only a small fraction of frames and with a fair-to-moderate (at both patient and lesion levels) agreement between AI-TCFA and core laboratory-TCFA (CL-TCFA).

Despite these encouraging scenarios, at present, the automation of atherosclerotic plaque assessment is largely limited to segmentation tasks. Deep learning-based methods enable highly accurate plaque delineation within milliseconds, enabling near-real-time processing, which is particularly important in the context of intravascular imaging [49]. Moreover, despite the promising potential of artificial intelligence in coronary plaque imaging, evidence from large-scale prospective randomized controlled trials remains limited. While AI may not require such validation for purely technical tasks, applications that influence clinical decision-making warrant further evaluation. Additional efforts are needed to ensure interpretability, standardization, and assessment of clinical impact before these tools can be widely implemented in routine practice [49].

## 3. Clinical Impact of ICI Plaque Characterization

Intracoronary imaging techniques have significantly expanded the diagnostic capabilities in ischemic heart disease [50]. Beyond anatomical assessment, these modalities allow for detailed characterization of plaque morphology and pathobiology, offering crucial insights into the mechanisms of ACS [51] and the identification of high-risk plaques [6]. Furthermore, intracoronary imaging has enhanced the understanding of dynamic processes such as plaque healing [52], contributing to a more comprehensive view of coronary atherosclerosis progression and stabilization. Finally, ICI may play a crucial role to guide management of high-risk plaques [53].

**Identification of mechanisms underlying acute coronary syndromes**

Beyond the identification of a culprit lesion, intracoronary imaging enables detailed characterization of plaque pathology, allowing for the distinction among three principal phenotypes: plaque rupture, plaque erosion, and calcified nodules [54]. Each phenotype carries distinct clinical implications, both in terms of prognosis and therapeutic strategy [54].

Plaque rupture (PR), characterized by fibrous cap disruption and exposure of the necrotic lipid core, represents the most common substrate of ACS, accounting for up to 60% of all ACS cases [23,55]. This mechanism is typically associated with thin-cap fibroatheroma (TCFA) plaques, characterized by a fibrous cap thickness of less than 65 μm; large lipid-rich plaques (lipid arc > 90°); and prominent macrophage infiltration (MØI)—features that are only identifiable by OCT [23,56,57]. Importantly, the identification of a ruptured culprit plaque carries significant prognostic implications, as it is associated with a higher incidence of long-term major adverse cardiac events (MACE) compared to culprit plaques with an intact fibrous cap (IFC), reflecting a more aggressive atherosclerotic phenotype and emphasizing the need for intensified secondary prevention strategies [58]. In fact, OCT studies have demonstrated that plaque rupture is often not an isolated event but rather a manifestation of panvascular vulnerability, as patients with acute coronary syndromes frequently present with multiple nonculprit plaques exhibiting features of high-risk morphology, suggesting a predisposition to plaque destabilization [59,60].

Plaque rupture (PR), characterized by fibrous cap disruption and exposure of the necrotic lipid core, represents the most common substrate of ACS, accounting for up to 60% of all ACS cases [23,55]. This mechanism is typically associated with thin-cap fibroatheroma (TCFA) plaques, characterized by a fibrous cap thickness of less than 65 μm; large lipid-rich plaques (lipid arc > 90°); and prominent macrophage infiltration (MØI)—features that are only identifiable by OCT [23,56,57]. Importantly, the identification of a ruptured culprit plaque carries significant prognostic implications, as it is associated with a higher incidence of long-term major adverse cardiac events (MACE) compared to culprit plaques with an intact fibrous cap (IFC), reflecting a more aggressive atherosclerotic phenotype and emphasizing the need for intensified secondary prevention strategies [58]. In fact, OCT studies have demonstrated that plaque rupture is often not an isolated event but rather a manifestation of panvascular vulnerability, as patients with acute coronary syndromes frequently present with multiple nonculprit plaques exhibiting features of high-risk morphology, suggesting a predisposition to plaque destabilization [59,60].

Furthermore, systemic inflammation, as reflected by elevated high-sensitivity C-reactive protein (hs-CRP) levels, has been shown to be associated with the presence of multiple plaque ruptures in patients presenting with acute myocardial infarction, which in turn portends a worse prognosis [61,62,63], underscoring the need for therapeutic approaches following ACS aimed at reducing residual inflammatory risk to prevent future cardiovascular events [64,65].

Conversely, about 40% of ACS events arise from non-ruptured plaques, characterized by an IFC, which include mechanisms such as plaque erosion (PE) and calcified nodules (CN) [7,66].

Plaque erosion (PE), defined by the presence of thrombus overlying an IFC without evident cap disruption [67], accounts for a substantial proportion of ACS cases (around one-third) [68,69], particularly among younger individuals, women, and patients with fewer traditional cardiovascular risk factors [70,71]. Although OCT offers high resolution (10–15 μm), it cannot directly visualize the endothelial monolayer (1–5 μm) [71]. Since endothelial loss is the pathological hallmark of plaque erosion, this limitation prevents its direct identification by OCT, prompting the development of dedicated intracoronary imaging-based classification algorithms [71]. Jia et al. described “definite OCT-erosion” as the presence of attached thrombus overlying an intact and clearly visualized plaque, whereas “probable OCT-erosion” is diagnosed based on luminal surface irregularities without thrombus or plaque attenuation by thrombus without adjacent superficial lipid or calcification [7]. OCT-based criteria do not always require the presence of attached thrombus, as they take into account the potential effects of prior treatment with antithrombotic therapy and/or thrombolysis [7].

OCT studies have shown that eroded culprit plaques, compared to those with PR, are associated with a lower frequency of lipid-rich plaques, a thicker fibrous cap, a smaller lipid arc, and less severe lumen stenosis [7], along with fewer features of widespread coronary plaque vulnerability [7,72]. The OPTICO-ACS (OPTIcal-COherence Tomography in Acute Coronary Syndrome) study demonstrated that patients with ACS due to PE have a more favorable prognosis compared to those with PR and exhibit a distinct inflammatory profile characterized by lower baseline expression of interleukin-6 (IL-6) and interleukin-1β-related proteins, suggesting that lesion phenotype may guide future personalized anti-inflammatory strategies in ACS management [73]. However, patients with OCT-defined plaque erosion and coexistent MØI at the culprit site exhibit a more severe atherosclerotic profile and greater disease burden, and this infiltration predicts worse long-term outcomes, highlighting the prognostic importance of intracoronary imaging for risk stratification and management planning [68]. Of note, plaque rupture (PR) and plaque erosion (PE) have been demonstrated to play a central role not only in atherosclerotic coronary artery disease but also in atherosclerotic plaque instability driven myocardial infarction with non-obstructive coronary arteries (MINOCA). The ICI technique, and above all OCT, could play a central role also in this setting since it has been demonstrated that understanding the underlying MINOCA etiology with a tailored diagnostic (and therapeutic) approach [74] leads to an improvement in angina-related health status if compared to the standard approach [75,76,77].

Calcified nodules (CN) represent a less common cause of acute coronary syndromes, accounting for fewer than 10% of culprit lesions [78,79]. They are characterized by eruptive, nodular calcifications that disrupt the fibrous cap and protrude into the coronary lumen, promoting thrombus formation [80,81]. The identification of CN by OCT or IVUS holds prognostic significance, as culprit lesions with calcified nodules are associated with a higher risk of recurrent adverse events compared to those with PR or PE, particularly in terms of target lesion revascularization (TLR) [82]. This worse prognosis is largely attributable to the mechanical challenges posed by heavily calcified lesions, which complicate optimal stent expansion and increase the risk of residual stenosis or stent underexpansion, factors known to negatively affect long-term outcomes. For example, the challenge posed by CN remains far from resolved, as the ongoing VICTORY trial (NCT05346068) comparing various contemporary lesion-preparation devices has not provided encouraging signals.

**Detection of high-risk plaques**

Only a subset of patients with high-risk plaques ultimately develops an acute coronary syndrome. This largely depends on secondary factors—especially systemic ones—which, acting as a kind of “second hit,” eventually lead to the acute destabilization of the plaque [6,83]. These factors include macrophage infiltration, the presence of a lipid-rich core, plaque burden, a thin fibrous cap (TCFA), systemic inflammation, impaired healing, alterations in local hemodynamic patterns, and the extent of the subtended myocardium at risk [6]. Overall, the interplay of these factors defines what is commonly referred to as the “high-risk patient,” who is predisposed to developing an acute coronary syndrome arising from vulnerable plaques [6]. For this reason, not only a plaque risk evaluation but also a patient level risk-stratification is crucial to understand which individual is more at risk of short-term coronary events [84].

The term “high-risk plaque” should therefore be applied to describe a lesion with an increased propensity to progress to clinically relevant thrombosis and to precipitate ACS (most commonly ST-segment elevation MI or type 1 non–ST-segment elevation MI) or cardiac death [6]. This designation is based on its morphology, overall burden, biological activity, intrinsic thrombogenicity, and anatomical location, with proximal coronary plaques being more involved in the mechanism of rupture, especially if located in proximal left anterior descending (LAD) [85,86,87,88]. Among these secondary factors, thin-cap fibroatheroma (TCFA), characterized by a lipid-rich core (lipid arc > 90°) covered by a thin fibrous cap (thickness ≤ 65 µm) [22], represents the classical example of rupture-prone plaque and has long been regarded as the archetype of the “vulnerable plaque” [89]. Importantly, while TCFAs are more frequently found in plaques with mild-to-moderate stenosis, those located within severely stenotic segments (>70% diameter stenosis) tend to exhibit a higher prevalence of high-risk features—such as thinner fibrous caps, greater plaque burden, increased microvascular density, and the presence of cholesterol crystals—making them more susceptible to rupture and subsequent clinical events [56].

OCT is nowadays commonly considered the intracoronary imaging technique of choice for high-risk plaque detection since it allows for the most detailed characterization of features associated with plaque vulnerability, including TCFA, lipid arch, large lipid pools, macrophage infiltration, and microcalcifications, as well as thrombosis precursors, which would be overlooked with IVUS or NIRS/IVUS imaging modalities [6,7,8].

In this setting, IVUS could be considered complementary to OCT since it reveals high plaque burden (>70%) and positive vessel remodeling, both indicators of increased plaque vulnerability [56].

Various large prospective studies supported the prognostic relevance of OCT-derived high-risk plaque features.

Among these, a precursor study was Xing et al.’s one, which first demonstrated that the detection of lipid-rich plaques in nonculprit segments of the target vessel by OCT predicts an elevated risk of subsequent nonculprit major adverse cardiac events, predominantly due to revascularization for recurrent ischemia (RR: 2.06 [95% CI: 1.05–4.04]; *p* = 0.036). Patients with plaques characterized by greater lipid length, a broader lipid arc, and more severe stenosis were identified as being at a higher risk for future cardiac events [90].

Some years later, the CLIMA (relationship between coronary plaque morphology of the left anterior descending artery and 12 months clinical outcome) study [91] showed that the simultaneous presence of four high-risk OCT features in the proximal left anterior descending artery—namely, minimum lumen area < 3.5 mm^2^, fibrous cap thickness < 75 μm, lipid arc > 180°, and MØI—was independently associated with a significantly increased risk of cardiac death and target-segment myocardial infarction at 12 months (HR 7.54; 95% CI 3.1–18.6; *p* < 0.001). The 5-year follow-up of the same study tried to assess the long-term prognostic implication of high-risk plaques [92]. At baseline, all four OCT criteria were present in 3.6% of patients, and their combined presence was strongly and independently associated with the primary endpoint at 5-year follow-up (adjusted HR 4.33; median follow-up 1.825 days, IQR 1.137–1.825). Patients meeting all four criteria also showed substantially higher risks of cardiac death (HR 3.73) and target segment myocardial infarction (TS-MI) (HR 7.02) compared with those without these features. This association with the primary endpoint persisted even after adjustment for high-intensity lipid-lowering therapy (adjusted HR 2.94). In addition, the presence of any TCFA, identified in 18.3% of patients, also predicted cardiac death and/or TS-MI [92].

The COMBINE OCT-FFR (thin-cap fibroatheroma predicts clinical events in diabetic patients with normal fractional flow reserve) trial [93] focused on diabetic patients with angiographically intermediate, FFR-negative lesions. Among these, the presence of OCT-detected TCFA identified a subgroup with a markedly higher incidence of MACE (13.3% and 3.1%; HR 4.65; 95% CI 1.99–10.89; *p* < 0.001) despite the absence of flow-limiting epicardial lesions [93,94].

More recently, a study by Jiang et al. [95] evaluated 883 patients with myocardial infarction who underwent OCT of all three major epicardial arteries during primary percutaneous coronary intervention. The primary endpoint was defined as the composite of cardiac death, nonculprit lesion-related nonfatal myocardial infarction and unplanned coronary revascularization. Patients were followed for up to 4 years, with a median follow-up of 3.3 years. Over a 4-year follow-up, the cumulative incidence of the primary endpoint was 7.2%. At the patient level, TCFA (adjusted HR: 3.05; 95% CI: 1.67–5.57) and a minimal lumen area (MLA) < 3.5 mm^2^ (adjusted HR: 3.71; 95% CI: 1.22–11.34) emerged as independent predictors of the primary endpoint. At the lesion level, nonculprit lesions that subsequently caused events were not angiographically severe at baseline. Both TCFA (adjusted HR: 8.15; 95% CI: 3.67–18.07) and MLA < 3.5 mm^2^ (adjusted HR: 4.33; 95% CI: 1.81–10.38) were associated with future events arising from individual lesions. The coexistence of TCFA and MLA < 3.5 mm^2^ conferred a particularly elevated risk, allowing for effective identification of patients susceptible to the composite outcome of cardiac death and nonculprit lesion-related nonfatal myocardial infarction.

Recent evidence has highlighted the phenomenon of plaque healing, a reparative response following minor, often subclinical, plaque disruptions [52]. After plaque disruption, thrombus formation triggers smooth muscle cell migration and extracellular matrix deposition, forming layered plaques that restore vascular integrity and prevent further thrombosis [52,96]. Impaired plaque healing is considered a “second hit” in ACS: intact healing stabilizes lesions, whereas defective repair promotes thrombosis and clinical events [97].

Healed plaque represents a common phenomenon: Shimokado et al. reported a 77% prevalence in chronic coronary syndrome patients comparing OCT with histology [98], while Burke et al. identified healed plaques in 61% of sudden cardiac death cases, particularly in patients with dyslipidaemia or diabetes, correlating with higher stenosis [99].

On OCT, healed coronary plaques (HCPs) appear as one or more signal-rich, heterogeneous layers over disrupted caps, correlating well with histopathology [52]. These lesions develop thicker fibrous caps and are less rupture-prone, though matrix accumulation may induce significant stenosis and stable angina [96].

In conclusion, intracoronary imaging not only aids in identifying vulnerable plaques but also offers a dynamic view of the natural history of coronary atherosclerosis, with important implications for future preventive therapies.

**Management of high-risk plaques**

**Figure 2:**
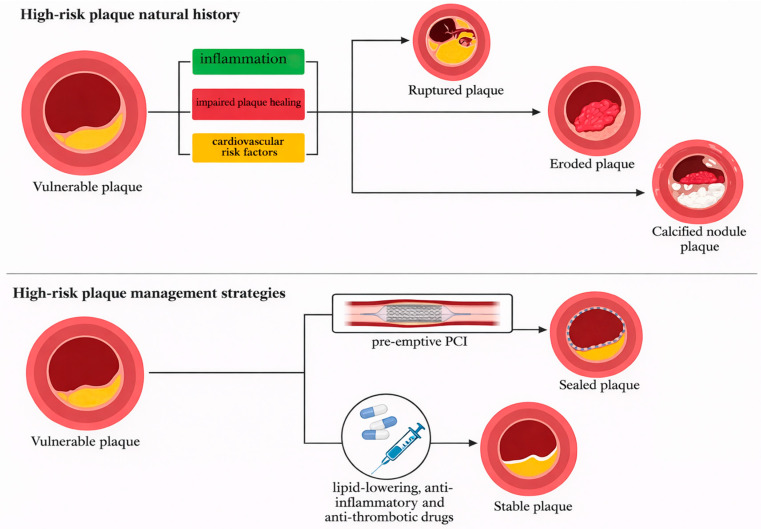
Provides an overview of current strategies for the management of vulnerable coronary plaques, integrating both invasive and pharmacological approaches. The invasive strategy consists of preventive percutaneous coronary intervention through plaque sealing, aimed at stabilizing high-risk plaques. The pharmacological approach focuses on systemic therapy-including high-intensity lipid-lowering, anti-inflammatory, and antithrombotic drugs-to promote plaque stabilization and regression. This figure contrasts the natural history of vulnerable plaques, including rupture, erosion, and calcified nodules, with the targeted interventions designed to prevent progression to acute coronary syndromes. Abbreviations: PCI = Percutaneous Coronary Intervention.

The ideal management of high-risk plaque is still a matter of debate. Recent advances in pharmacotherapy demonstrated the key role of modern preventive drug therapies to stabilize coronary plaques. On the other hand, the use of PCI to passivate high-risk plaques remains attractive and tested in several trials. A comprehensive list of clinical trials regarding medical and interventional therapy for vulnerable plaques can be found in Table 2. A conceptual schematic representation of medical and interventional therapy for vulnerable plaques can be found in Figure 2.

**(1)**  **Pharmacological approach**

Aggressive medical therapy remains the standard of care for high-risk plaques, achieving measurable improvements in plaque morphology and stability. The “three pillars” on which relies the pharmacological approach to vulnerable plaques are lipid-lowering drugs, anti-inflammatory drugs, and anti-thrombotic drugs.

  **(A)**  **Lipid-lowering drugs**

It is well established that lipid-lowering therapy not only reduces plaque volume but also promotes plaque stabilization [100].

One of the first intracoronary imaging-based studies, which showed the beneficial effect of statins of plaque volume and morphology, dates back to 1997: Takagi et al. managed to demonstrate that the administration of pravastatin reduces IVUS-detected progression of coronary artery plaque in patients with elevated total cholesterol levels [101]. Since then, a great number of randomized controlled trials (RCTs) substantially brought similar results.

The ASTEROID (A Study to Evaluate the Effect of Rosuvastatin on Intravascular Ultrasound-Derived Atheroma Regression) trial [102], by Nissen et al., demonstrated that very high-intensity statin therapy with rosuvastatin 40 mg/day achieved a mean low-density lipoprotein cholesterol (LDL-c) of 60.8 mg/dL and increased high-density lipoprotein cholesterol (HDL-c) by 14.7%, leading to significant regression of atherosclerosis as assessed by IVUS measures of disease burden. These findings indicate that lowering LDL-c below current guideline targets, particularly when accompanied by substantial HDL-c elevation, can promote atherosclerosis regression in patients with coronary artery disease.

The IBIS-4 (The Integrated Biomarkers and Imaging Study 4) trial [103] showed similar outcomes: among 103 ST-elevation myocardial infarction (STEMI) patients, high-intensity rosuvastatin (40 mg/day) over 13 months significantly reduced LDL-c from 3.29 to 1.89 mmol/L and modestly increased HDL-c from 1.10 to 1.20 mmol/L. Serial IVUS imaging of 146 non-infarct-related arteries in 82 patients showed a 0.9% reduction in percent atheroma volume, with 74% of patients demonstrating regression in at least one artery. Necrotic core proportion and thin-cap fibroatheroma counts remained unchanged. These findings indicate that intensive statin therapy effectively reduces overall atherosclerotic burden in STEMI patients, though plaque composition may remain largely unaltered, with potential long-term clinical benefits.

The YELLOW (Reduction in Yellow Plaque by Aggressive Lipid-Lowering Therapy) trial [104] randomized 87 patients with multivessel coronary artery disease undergoing PCI and at least one additional obstructive nontarget lesion (FFR ≤ 0.80) to receive either intensive lipid-lowering therapy with rosuvastatin 40 mg/day or standard care. Nontarget lesions were assessed at baseline and after 7 weeks using FFR, NIRS, and IVUS. Intensive therapy resulted in a significantly greater reduction in maximal 4 mm lipid-core burden index compared with standard therapy, even after adjustment for baseline differences.

The EASY-FIT (effect of atorvastatin therapy on fibrous cap thickness in coronary atherosclerotic plaque as assessed by optical coherence tomography) study [105] randomized 70 patients with unstable angina and untreated dyslipidaemia to receive atorvastatin 20 mg/day or 5 mg/day, with OCT used to assess intermediate nonculprit lesions at baseline and 12 months. LDL-c levels were significantly lower with 20 mg/day compared with 5 mg/day (69 vs. 78 mg/dL; *p* = 0.039). High-dose therapy produced a greater increase in fibrous cap thickness (69% vs. 17%; *p* < 0.001), which correlated with reductions in LDL-c, oxidized LDL, hs-CRP, matrix metalloproteinase-9 (MMP-9), and OCT-derived macrophage grade, indicating that intensive statin therapy not only lowers lipid levels but also promotes plaque stabilization by thickening the fibrous cap and reducing local inflammatory activity.

In terms of different statin atheroma volume-reducing power, it has been demonstrated that high-dose Atorvastatin is more effective than moderate-dose Pravastatin in reducing atheroma volume after 18 months (REVERSAL trial) [106] and that high-dose Atorvastatin and Rosuvastatin own a similar IVUS-evaluated atheroma volume-reducing power, although the latter demonstrated a benefit with respect to normalized total atheroma volume (SATURN trial) [107].

In the era of Proprotein Convertase Subtilisin/Kexin Type 9 inhibitors (PCSK9-i), some studies tried to assess the atheroma-reducing effect of those drugs added on top of statins.

One of the first trials published was the GLAGOV (Global Assessment of Plaque Regression With a PCSK9 Antibody as Measured by Intravascular Ultrasound) trial [108]: a study of 968 participants with angiographic coronary disease, which randomized patients to monthly subcutaneous evolocumab at 420 mg (*n* = 484) or placebo (*n* = 484) for 76 weeks, in addition to statins. Serial IVUS imaging was available for 846 patients. Evolocumab achieved significantly lower mean LDL-c compared with the placebo (36.6 vs. 93.0 mg/dL; *p* < 0.001). The percent atheroma volume decreased by 0.95% with evolocumab versus a 0.05% increase with placebo (difference −1.0%; *p* < 0.001). Normalized total atheroma volume declined by 5.8 mm^3^ with evolocumab and 0.9 mm^3^ with the placebo (difference −4.9 mm^3^; *p* < 0.001). Plaque regression occurred more frequently with evolocumab (64.3% vs. 47.3% for percent atheroma volume; 61.5% vs. 48.9% for total atheroma volume; both *p* < 0.001). These results demonstrate that PCSK9 inhibition with evolocumab, when added to statin therapy, achieves substantial LDL-c reduction and promotes coronary plaque regression.

The subsequent HUYGENS (High-Resolution Underlying-Plaque Assessment With Evolocumab Using Intravascular Ultrasound) trial [109] showed that PCSK9-i treatment was associated with greater increases in minimum fibrous cap thickness evaluated by OCT at 52 weeks follow-up (42.7 vs. 21.5 µm; *p* = 0.015) and more pronounced reductions in lipid arc extension (−57.5% vs. −31.4%; *p* = 0.04) compared with placebo.

More recently, the PACMAN-AMI (Effects of the PCSK9 Antibody Alirocumab on Coronary Atherosclerosis in Patients With Acute Myocardial Infarction) trial [110] demonstrated that early administration of PCSK9-i on top of statins enhances plaque regression and stabilization after AMI. This randomized and double-blind trial enrolled 300 patients with acute myocardial infarction undergoing PCI. Participants received biweekly alirocumab (150 mg) or placebo, initiated within 24 h post-PCI, in addition to high-intensity statin therapy, for 52 weeks. Serial IVUS, NIRS, and OCT of non-infarct arteries demonstrated greater regression of percent atheroma volume (−2.13% vs. −0.92%; *p* < 0.001), larger reductions in lipid core burden index (−79.4 vs. −37.6; *p* = 0.006), and greater increases in fibrous cap thickness (62.7 µm vs. 33.2 µm; *p* = 0.001) with alirocumab compared to placebo.

Of note, a more recent trial, the ODYSSEY-J IVUS (Effect of Alirocumab on Coronary Atheroma Volume in Japanese Patients With Acute Coronary Syndrome) trial [111], represents a deviation from the amount of evidence in favor of PCSK9-i drugs since it demonstrated that in patients with ACS and statin-resistant hypercholesterolemia, alirocumab produced a numerically greater reduction in total atheroma volume at week 36 compared with standard therapy, although the difference did not reach statistical significance.

As for other lipid-lowering drugs, both ezetimibe and icosapent-ethyl (IPE) demonstrated a beneficial effect on the coronary plaque volume reduction, when added on top of statins.

With respect to ezetimibe, PRECISE-IVUS (Plaque Regression With Cholesterol Absorption Inhibitor or Synthesis Inhibitor Evaluated by Intravascular Ultrasound) trial [112] was a prospective, randomized, multicenter study, which enrolled 202 patients undergoing PCI who were assigned to atorvastatin alone or atorvastatin plus ezetimibe (10 mg daily), with statin uptitrated to achieve LDL-c < 70 mg/dL. Serial IVUS at baseline and 9–12 months assessed plaque response: Combination therapy achieved lower LDL-c levels than statin alone (63.2 vs. 73.3 mg/dL; *p* < 0.001) and demonstrated superior reduction in percent atheroma volume (−1.4% vs. −0.3%; *p* = 0.001). A higher proportion of patients receiving dual therapy experienced plaque regression (78% vs. 58%; *p* = 0.004).

As regards icosapent-ethyl, the EVAPORATE (Effect of icosapent-ethyl on progression of coronary atherosclerosis in patients with elevated triglycerides on statin therapy) trial [113], a randomized, double-blind, placebo-controlled study, showed that icosapent-ethyl (4 g/day) added to statin therapy—in patients with documented atherosclerosis—attenuates coronary plaque progression beyond standard statin therapy, particularly for overall and fibrous plaque components. The pre-specified primary endpoint was the change in low-attenuation plaque (LAP) volume at 18 months: IPE treatment produced a 17% reduction in LAP volume versus a >2-fold increase (+109%) in the placebo group (*p* = 0.0061). Significant between-group differences were also observed for fibrous and fibrofatty (FF) plaque volumes, which regressed with IPE but progressed with placebo (*p* < 0.01).

  **(B)**  **Anti-inflammatory drugs**

As for anti-inflammatory drugs, one of the most used ones in a coronary artery disease (CAD) setting is colchicine, especially considering that 2024 ESC chronic coronary syndrome guidelines endorse the use of low-dose colchicine (0.5 mg daily) to reduce myocardial infarction, stroke, and the need for revascularization (Class of Recommendation IIa, Level of Evidence A) [28]. Despite this, its effect on atheroma volume is still debated, and the routine use for colchicine in ACS remains argued [65,114,115]. Indeed, the COLOCT study and the COCOMO-ACS trial brought discordant results.

The first one, the Colchicine on Coronary Plaque Stability Trial Assessed by Optical Coherence Tomography (COLOCT) trial [116], was a single-center, double-blind trial that enrolled 128 ACS patients with lipid-rich plaques identified by OCT and randomized them to colchicine 0.5 mg/day or placebo for 12 months. Colchicine significantly increased minimal fibrous cap thickness (87.2 vs. 51.9 μm; *p* = 0.006), reduced average lipid arc (−35.7° vs. −25.2°; *p* = 0.004), and decreased macrophage angular extension (−14.0° vs. −8.9°; *p* = 0.044) compared with the placebo, showing a colchicine-positive effect of plaque vulnerability.

On the other hand, the optical coherence tomography assessment of the impact of colchicine on nonculprit coronary plaque composition after myocardial infarction (COCOMO-ACS) trial [117], a double-blind, placebo-controlled trial, randomized 64 patients with non-ST-elevation MI to colchicine at 0.5 mg/day or placebo for a median of 17.8 months, showing no significant effect of colchicine on minimum fibrous cap thickness or maximum lipid arc overall alongside standard therapy at serial OCT evaluation of nonculprit plaques.

The discordant findings likely reflect differences in study design and focus: COLOCT’s large sample and clinical endpoints versus COCOMO’s small imaging-based approach. Colchicine’s anti-inflammatory mechanism (downregulating IL-6/CRP) could reduce events even if measurable plaque morphology changes lag behind. Further studies could clarify how colchicine’s systemic effects translate into plaque stabilization over time.

  **(C)**  **Antithrombotic drugs**

Up to the current date, there are no statistically strong data about antithrombotic drugs’ positive effects on atheroma volume or composition [118].

Nevertheless, antithrombotic drugs still have a role in vulnerable plaque management since recent evidence shows they could play a relevant part in plaque erosion.

Regarding the effective anti-thrombotic therapy without stenting, the intravascular optical coherence tomography-based management in plaque erosion study (the EROSION study) [119] have suggested that a subset of ACS patients presenting with plaque erosion, residual stenosis < 70%, and Thrombolysis In Myocardial Infarction (TIMI) grade 3 flow may be safely managed with intensive antithrombotic therapy alone without the need for immediate stent implantation. Indeed, the single-arm EROSION study [119], with 78.3% of patients treated conservatively with antithrombotic therapy alone, achieved the primary endpoint, defined as a >50% reduction in thrombus volume after one month of antithrombotic therapy, with a low incidence of MACE. The subsequent randomized EROSION III trial [120] explored the broader applicability of OCT-guided management of ACS based on pathophysiology, reinforcing the concept that selected patients with non-ruptured culprit lesions may benefit from a stent-free strategy. This strategy has the potential to minimize the risks associated with stent implantation, such as stent thrombosis and in-stent restenosis, while preserving vessel physiology and reducing the need for prolonged dual antiplatelet therapy.

**(2)**  **Pre-emptive percutaneous coronary intervention**

Over the past decades, most efforts to reduce the risk of adverse cardiovascular events have concentrated on systemic therapies aimed at slowing or stabilizing atherosclerotic disease. More recently, however, interventional approaches have been proposed to directly reinforce plaques identified as high risk [121]. The rationale is that a local device—whether a metallic stent or a bioresorbable vascular scaffold (BVS)—could act as a physical shield, covering vulnerable structures and triggering neointimal proliferation that functions as a “neo-cap,” thereby reducing the probability of rupture or erosion [122]. This concept, often referred to as preventive stenting or plaque sealing, departs from conventional PCI by targeting lesions not for flow limitation or acute presentation, but for their biological instability [6,123,124].

Sealing plaques pre-emptively is not without challenges. Device implantation in this context may carry risks of distal embolization, peri-procedural myocardial infarction, and longer-term complications such as restenosis or stent thrombosis [6]. Concerns have also been raised from pathology studies showing that metallic struts within necrotic cores may fail to achieve complete endothelial coverage, potentially serving as niduses for late thrombotic events [124].

In this regard, BVS have generated particular interest. Unlike metallic devices, these scaffolds are gradually resorbed and replaced by connective tissue and smooth muscle, leaving behind a remodeled vessel wall. This process not only avoids the issue of permanent metallic persistence but also supports the development of a stable fibrotic layer overlying thin-cap fibroatheromas [124].

The PROSPECT-ABSORB study [125], embedded within the PROSPECT II [126] program, was the first randomized trial designed to test focal preventive therapy of high-risk but non-obstructive coronary plaques. After successful PCI of culprit lesions in patients with myocardial infarction, three-vessel imaging with combined NIRS-IVUS identified nonculprit plaques characterized by ≥65% burden. A total of 182 patients were assigned to either implantation of a BVS plus guideline-directed medical therapy (GDMT) or GDMT alone. The principal efficacy endpoint was the IVUS-derived minimum lumen area (MLA) at approximately 25 months, while safety was assessed through target lesion failure at 24 months, and lesion-related MACEs were evaluated over longer-term follow-up. At imaging follow-up, BVS-treated lesions demonstrated substantially larger luminal dimensions compared with those managed conservatively (mean MLA 6.9 ± 2.6 mm^2^ vs. 3.0 ± 1.0 mm^2^; *p* < 0.001). BVS therapy was also associated with favorable morphologic changes, including neo-cap formation and plaque delipidation. Event rates were low across both groups: target lesion failure at 24 months was nearly identical (4.3% vs. 4.5%), while lesion-related MACE at four years was numerically lower with BVS (4.3% vs. 10.7%; OR 0.38, 95% CI 0.11–1.28), though not statistically significant. PROSPECT-ABSORB trial demonstrated that prophylactic BVS implantation on angiographically mild but high-risk plaques is feasible, induces positive remodeling, and may reduce lesion-related events. However, the low incidence of clinical endpoints under modern medical therapy highlights the necessity for larger, adequately powered studies before this approach can be adopted in routine practice.

The subsequent PREVENT (Preventive percutaneous coronary intervention versus optimal medical therapy alone for the treatment of vulnerable atherosclerotic coronary plaques) trial [127] provided the first randomized evidence supporting a therapeutic benefit of preventive PCI targeting angiographically intermediate, non-flow-limiting but vulnerable plaques. Vulnerable lesions were defined as focal, FFR-negative plaques (>50% diameter stenosis) exhibiting at least two imaging-derived high-risk features: minimum lumen area (MLA) < 4.0 mm^2^ by IVUS or OCT; plaque burden > 70% by IVUS; lipid-rich plaque by NIRS; or the presence of a TCFA by IVUS or OCT. Patients randomized to preventive PCI experienced a significantly lower incidence of the composite endpoint—including cardiac death, target-vessel myocardial infarction (MI), hospitalization for unstable angina, or ischemia-driven revascularization—compared with those managed with optimal medical therapy alone (0.4% vs. 3.4% at 2 years; 95% CI −4.4 to −1.8; *p* = 0.0003). However, the number needed to treat (NNT) to prevent one cardiac death or target-vessel MI was relatively high (87.7), suggesting limited absolute risk reduction in this population. Of note, only about 63% of patients in the medical therapy arm were receiving high-intensity statins or moderate-intensity statins plus ezetimibe at maximal follow-up, potentially underestimating the efficacy of optimized conservative management.

The SECRITT trial [123], instead, demonstrated the efficacy of self-expanding nitinol intracoronary device with low radial force (v-Shield device) in sealing the high-risk IVUS and optical coherence tomography-identified TCFA. This small randomized study of 23 patients with non-obstructive TCFA showed that v-Shield placement was safe and feasible. At six months, lesions showed increased fibrous cap thickness (from 48 µm to 201 µm), reduced stenosis, stable FFR, minimal late loss, and no device-related MACE. Neo-cap formation and improved stent apposition were observed.

New evidence in the complex puzzle of preventive PCI for vulnerable plaques will be brought by some ongoing studies such as the COMBINE-INTERVENE (Combined Ischemia and Vulnerable Plaque Percutaneous Intervention to Reduce Cardiovascular Events; NCT05333068) trial, the VULNERABLE (Treatment of Functionally Non-Significant Vulnerable Plaques in Patients With Multivessel ST-Elevation Myocardial Infarction; NCT05599061) trial, the INTERCLIMA (Interventional Strategy for Nonculprit Lesions With Major Vulnerability Criteria Identified by OCT in Patients With ACS; NCT05027984) trial, the FAVOR 5 AMI (Functional and Angiography-Derived Strain Guided Multivessel/Lesion Revascularization Strategy in Patients With Acute ST-Segment Elevation Myocardial Infarction; NCT05669222) trial, the RESTORE (Preventive Drug-Coated Balloon Angioplasty in Vulnerable Atherosclerotic Plaque Trial; NCT06365502) trial, the PASSIVATE-CAP (Passivation of Vulnerable Coronary Atherosclerotic Plaques; NCT06416813) trial, and the POLARSTAR trial (Polar Cryo-Energy Lesion Stabilization Research in Coronary Arteries Study Using Cryotherapy; NCT05600088).

**Table 2:** Summarizes the main studies on the pharmacological and interventional management of vulnerable plaques.

Study Name	Study Year	Study Design	Sample Size	Comparison	Primary Endpoint (and ICI Technique Used)	Result
Takagi et al. [101]	1997	Randomized	36 patients	Pravastatin and diet vs. diet only	Plaque progression (IVUS)	Reduced plaque progression with pravastatin
ASTEROID [102]	2006	Randomized	507 patients	Rosuvastatin 40 mg/day	Atheroma regression (IVUS)	Significant atherosclerosis regression with rosuvastatin
IBIS-4 [103]	2014	Prospective	103 patients	Rosuvastatin 40 mg/day	Percent atheroma volume (IVUS)	0.9% reduction in percent atheroma volume
YELLOW [104]	2013	Randomized	87 patients	Rosuvastatin 40 mg/day vs. standard care	Lipid-core burden index (NIRS/IVUS)	Greater reduction in lipid index with intensive therapy
EASY-FIT [105]	2014	Randomized	70 patients	Atorvastatin 20 mg/day vs. 5 mg/day	Fibrous cap thickness (OCT)	Greater increase in cap thickness with higher dose
REVERSAL [106]	2004	Randomized	654 patients	High-dose Atorvastatin vs. moderate-dose Pravastatin	Percent atheroma volume (IVUS)	Atorvastatin more effective in reducing percent atheroma volume
SATURN [107]	2011	Randomized	1039 patients	High-dose Atorvastatin vs. high-dose Rosuvastatin	Total and percent atheroma volume (IVUS)	Similar reduction; benefit on normalized total atheroma volume with rosuvastatin
GLAGOV [108]	2016	Randomized	968 patients	Evolocumab 420 mg vs. placebo + statins	Percent atheroma volume (IVUS)	Significant reduction with evolocumab
HUYGENS [109]	2022	Randomized	161 patients	Evolocumab vs. placebo	Minimum fibrous cap thickness (OCT)	Greater increase with evolocumab
PACMAN-AMI [110]	2022	Randomized	300 patients	Alirocumab 150 mg + statins vs. placebo + statins	Plaque regression (IVUS, NIRS, OCT)	Greater regression with alirocumab
ODYSSEY-J IVUS [111]	2019	Randomized	206 patients	Alirocumab + statins vs. standard therapy + statins	Total atheroma volume (IVUS)	Numerically greater reduction, not statistically significant
PRECISE-IVUS [112]	2015	Randomized	202 patients	Atorvastatin + ezetimibe vs. atorvastatin alone	Percent atheroma volume (IVUS)	Superior reduction with combination therapy
EVAPORATE [113]	2020	Randomized	68 patients	Icosapent-ethyl 4 g/day vs. placebo + statins	Low-attenuation plaque (coronary computed tomography angiography)	IPE slowed plaque progression
COLOCT [116]	2024	Randomized	128 patients	Colchicine 0.5 mg/day vs. placebo	Plaque stability (OCT)	Increased cap thickness and reduced lipid arc
COCOMO-ACS [117]	2025	Randomized	64 patients	Colchicine 0.5 mg/day vs. placebo	Fibrous cap thickness and lipid arc (OCT)	No significant effect
EROSION [119]	2017	Prospective	405 patients	Intensive antithrombotic therapy without stenting	Thrombus volume reduction (OCT)	78.3% reached primary endpoint
PROSPECT-ABSORB [125]	2020	Randomized	182 patients	BVS + GDMT vs. GDMT alone	Minimum lumen area (IVUS)	Larger minimal luminal dimensions with BVS
PREVENT [127]	2024	Randomized	1606 patients	Preventive PCI vs. optimal medical therapy	Composite clinical endpoint (IVUS, vhIVUS, NIRS, NIRS/IVUS or OCT)	Lower incidence with preventive PCI
SECRITT [123]	2012	Randomized	23 patients	v-Shield device vs medical therapy	Safety and feasibility (vhIVUS, OCT)	Increased fibrous cap thickness; no major events

Abbreviations: ICI = Intracoronary Imaging; IVUS = Intravascular Ultrasound; NIRS/IVUS = Near-Infrared Spectroscopy/Intravascular Ultrasound; OCT = Optical Coherence Tomography; vhIVUS = Virtual Histology Intravascular Ultrasound; IPE = Icosapent Ethyl; BVS = Bioresorbable Vascular Scaffold; PCI = Percutaneous Coronary Intervention; GDMT = Guideline-Directed Medical Therapy.

## 4. Conclusions and Future Perspectives

The management of coronary high-risk plaques has progressively shifted from a sole focus on flow-limiting lesions to early identification and treatment of plaques prone to causing future acute coronary syndromes. Intracoronary imaging, particularly OCT, has been pivotal in this paradigm shift, providing the high-resolution assessment necessary to detect key morphological features of vulnerability, including thin-cap fibroatheroma, large lipid arcs, macrophage infiltration, and microcalcifications. This detailed plaque characterization allows clinicians to stratify risk more accurately and tailor therapeutic strategies.

Current evidence supports an initial strategy centered on intensive systemic medical therapy—particularly high-intensity lipid-lowering drugs and, in selected cases, anti-inflammatory agents—which stabilizes the coronary tree and promotes plaque regression. For a subset of lesions identified as high-risk, targeted interventional approaches, such as preventive PCI or bioresorbable vascular scaffold implantation, may offer additional protection by physically reinforcing vulnerable plaques, although this remains experimental and requires further validation in larger clinical trials. Importantly, imaging-guided approaches highlight that not all vulnerable plaques require immediate stenting, as illustrated by the successful conservative management of plaque erosion with antithrombotic therapy alone.

Looking forward, the integration of hybrid intracoronary imaging modalities, including NIRS-IVUS and other hybrid ICI techniques, along with artificial intelligence for automated plaque analysis, promises to further enhance risk stratification and procedural precision. Combining imaging data with computational simulations, hemodynamic assessments, and patient-specific characteristics (e.g., comorbidities, genetic profiles) could enable truly personalized intervention strategies. Moreover, the growing understanding of plaque healing and dynamic vascular remodeling underscores the need for longitudinal imaging to guide treatment and monitor response.

Clinically, these advances imply a move toward individualized, phenotype-driven management of coronary artery disease, where therapy is selected not solely based on stenosis severity but also on plaque biology and vulnerability. This approach may reduce the incidence of acute coronary events, optimize the use of invasive procedures, and improve long-term outcomes.

Nevertheless, challenges remain, including the limited positive predictive value of single imaging features and the need for larger trials to define the most effective combinations of medical and interventional therapies. Indeed, some studied showed than even when vulnerable features are identified, predictive accuracy is poor (for example, in the PROSPECT study [128], IVUS-derived parameters showed a limited ability to predict future culprit lesions, with a positive predictive value of only 18.2%). Moreover, there is no established way to combine imaging findings with overall patient risk yet. Lastly, the clinical benefit of pre-emptive treatment of vulnerable plaques remains uncertain.

In conclusion, intracoronary imaging-guided management of high-risk plaques, in combination with systemic therapy and emerging technological innovations, is shaping a more precise and personalized era in interventional cardiology. Continued research integrating hybrid imaging, artificial intelligence, and biological profiling will be crucial to fully realize the potential of preventive strategies and to translate high-resolution plaque characterization into a real clinical benefit.

## Figures and Tables

**Table 1:** Summarizes the technical principles, advantages, and limitations of IVUS, OCT, and NIRS [11,12].

Imaging Modality	Technical Principle	Advantages	Plaque Features Best Identified by the ICI Technique	Limitations
**OCT**	A catheter-mounted single-mode fiber and lens perform low-coherence interferometry on backscattered near-infrared light, producing real-time, high-resolution cross-sectional and volumetric images.	Highest resolution axial 10–20 μm and lateral 20–90 μm) among currently available intravascular modalities (10 times greater than IVUS). Detailed microstructural information (such as stent strut analysis). Superior for post-PCI optimization. Superior resolution for plaque morphology and vulnerability. Best for identifying ACS mechanism.	TCFA Macrophage infiltration (appearing as high-intensity bright spots) Calcification (appearing as sharply demarcated, signal-poor regions)	Limited penetration depth (2–3 mm). Requires blood clearance. Need for contrast.
**IVUS**	A catheter-mounted piezoelectric transducer emits ultrasound and captures real-time, 360°—cross-sectional images of coronary vessels.	Deeper tissue penetration (4–8 mm). No need for blood clearance. Superior for assessment of vessel dimensions and plaque burden. Best for larger vessels (such as left main). No need for contrast.	Plaque burden (plaque area/volume and lumen obstruction) Positive remodeling	Lower resolution compared to OCT. Artifact susceptibility.
**NIRS**	A catheter emits near-infrared light and analyzes the wavelength of the diffusely reflected light to determine the chemical composition of the target based on its unique spectral signature.	FDA-approved for detection and mapping of lipid-rich plaques (LRP). Can acquire data through stent struts and calcification. Provides a semiquantitative chemogram (lipid core burden index—LCBI) to assess lipid content. No need for contrast.	Lipid rich cores (appearing as yellow regions on chemogram)	Does not provide structural or 3D vessel imaging. Delivers solely semiquantitative measurements (no detailed morphology). Often integrated with IVUS to overcome its structural limitations.

Abbreviations: OCT = Optical Coherence Tomography; IVUS = Intravascular Ultrasound; NIRS = Near-Infrared Spectroscopy; TCFA = Thin-Cap Fibroatheroma; ACS = Acute Coronary Syndrome; PCI = Percutaneous Coronary Intervention; FDA = Food and Drug Administration; LRP = Lipid-Rich Plaque; LCBI = Lipid Core Burden Index.

## Data Availability

No new data were created or analyzed in this study. Data sharing is not applicable to this article.
